# Intravascular Large B-cell Lymphoma: A Report of Two Cases

**DOI:** 10.30699/ijp.2020.119590.2299

**Published:** 2020-07-16

**Authors:** Fereshteh Ameli, Fatemeh Nili Ahmad Abadi, Hana Saffar

**Affiliations:** 1 *Department of Pathology, Cancer Institute, Imam Khomeini Hospital Complex, Tehran University of Medical Sciences, Tehran, Iran*

**Keywords:** Extranodal large B-cell lymphoma, Intravascular lymphoma, Fever of Unknown Origin (FUO)

## Abstract

One of the rare variants of extranodal large B-cell lymphoma is intravascular large B-cell lymphoma (IVLBCL). Characteristics of IVLBCL include intraluminal selective proliferation of atypical lymphoid cells in small to medium-sized vessels. The etiologic of IVLBCL is unknown, but due to the growth pattern of this tumor, it is speculated that IVLBCL is caused by a defect in homing receptor of tumor cells. IVLBCL can involve any organ but central nervous system, lungs, and skin are the most involved sites. IVLBCL does not usually involve lymph nodes. IVLBCL mainly occurs in the middle aged to elderly population with a slight male predominance. Generally, IVLBCL is aggressive and rapidly fatal if left untreated.

We here reported two cases of IVLBCL who succumbed to the disease at initial phase of treatment to emphasize the difficulty in diagnosis of IVLBCL due to its exclusive intravascular growth pattern and fulminant clinical course.

## Introduction

Intravascular large B-cell lymphoma (IVLBCL), otherwise known as intravascular lymphomatosis or angiotropic lymphoma, is a rare variant of extranodal large B-cell lymphoma. In IVLBCL the atypical lymphoid cells float predominantly within the lumens of blood vessels especially capillaries, with no or few circulating neoplastic cells in the peripheral blood (1,2). The hallmark of diagnosis of IVLBCL is the remarkable degree of sparing of the surrounding tissue besides the absence of the tumoral cells in lymph nodes and the reticuloendothelial system (3). 

The underlying mechanism for this angiotropism is not clearly understood. It is hypothesized that the absence of adhesion molecules, including b1 integrin, (ICAM-1) and metalloproteinases on the tumoral cells can affect transvascular penetration of extracellular matrix for the tumor mass formation (3,4).

Two major clinical presentation patterns have been described for IVLBCL. A so-called classic form, mostly present in western countries, is characterized by symptoms related to the involvement of main organs, predominantly neural or cutaneous involvement. The Asian variant is a haemophagocytic syndrome-associated form, where patients present with multi-organ failure, hepatosplenomegaly, and pancytopenia. (1,2).

IVLBCL is an aggressive disease and has a poor prognosis. The three-year survival for the cutaneous-only form of IVLBCL is 56% and reduced to 22% if it spreads beyond the skin. IVLBCL can be rapidly fatal if left untreated (5).

The chemotherapy regime for IVLBCL includes rituximab plus cyclo-phosphamide, hydroxydaunorubicin, vincristine, and prednisone (R-CHOP). R-CHOP has significantly improved the prognosis of IVLBCL. However, relapse, particularly in the brain, is observed in a substantial proportion of patients, where the efficacy of R-CHOP is minimal (6).

In this case series, we described the clinicopathological findings of two patients with IVLBCL. To the best of our knowledge, this is the first case-series report seen in Iran.

##  Case Report

**Case 1**

A 69-year-old diabetic lady presented with fever of unknown origin (FUO) and pruritic skin lesions. On admission, hepatosplenomegaly was apparent, but no lymphadenopathy was detected. The laboratory examination revealed bicytopenia and increased levels of the liver enzymes. 

**Fig. 1:**
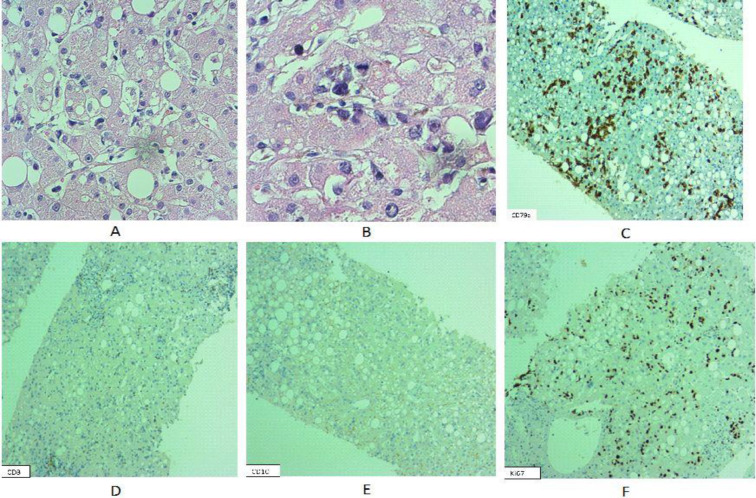
Histological and immunohistochemical features of intravascular large B cell lymphoma in the liver biopsy of the first case. A. H&E sections show an intra-sinusoidal infiltrate of atypical lymphoid cells in the liver (400x). B. The atypical cells are large with hyperchromatic nuclei, minimal cytoplasm, thick nuclear membrane, irregular nuclear contours and some with prominent nucleoli (H&E, 400x). The malignant lymphoid cells show strong CD79a immunostaining, but no CD3 or CD10 immunoreactivity (D, E). Ki67 highlights high proliferation index of intracapillary malignant lymphoid cells (F).

**Fig. 2:**
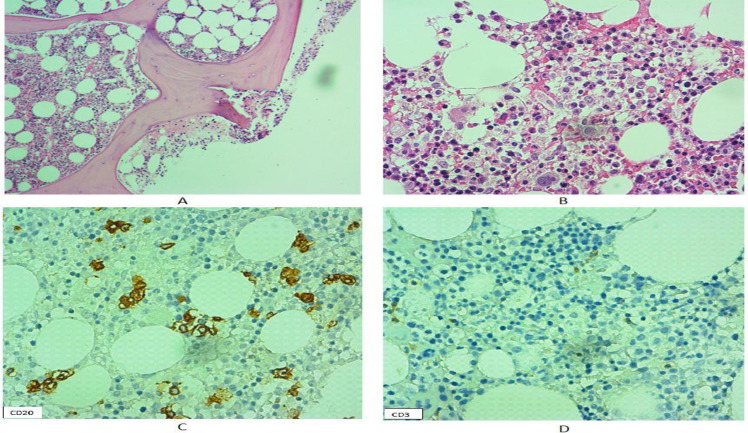
Histological and immunohistochemical features of intravascular large B cell lymphoma in bone marrow biopsy of the first case. A-B. H&E sections show no obvious evidence of bone marrow involvement (100x and 400x, respectively). C. CD20 immunoreactivity reveals the intravascular growth pattern of atypical B cells in sinusoidal spaces of bone marrow tissue. D. Negative immunoreactivity of tumoral cells for CD3

Histological analysis of the liver biopsy demonstrated a proliferation of atypical lymphoid cells with positive immunoreactivity for CD79a and CD20 in the small capillaries leading to the diagnosis of intravascular large B-cell lymphoma. No immunoreactivity for CD3, CD4, CD8, CD56, CD10 or BCL6 was detected (Figure 1 A-F). The patient underwent a bone marrow biopsy, but neither obvious tumoral involvement in hematoxylin and eosin stained sections nor hemophagocytosis in bone marrow aspiration were identified. Immunohistochemical study for CD20 on bone marrow sections highlighted intravascular malignant lymphoid cells, which was indicative of bone marrow involvement (Figure 2 A-D). Unfortunately, the patient’s condition rapidly worsened, with the development of multi-organ failure and loss of consciousness. She succumbed to the disease at initial phase of chemotherapy (within the first week of treatment).

**Case 2**

**Fig. 3:**
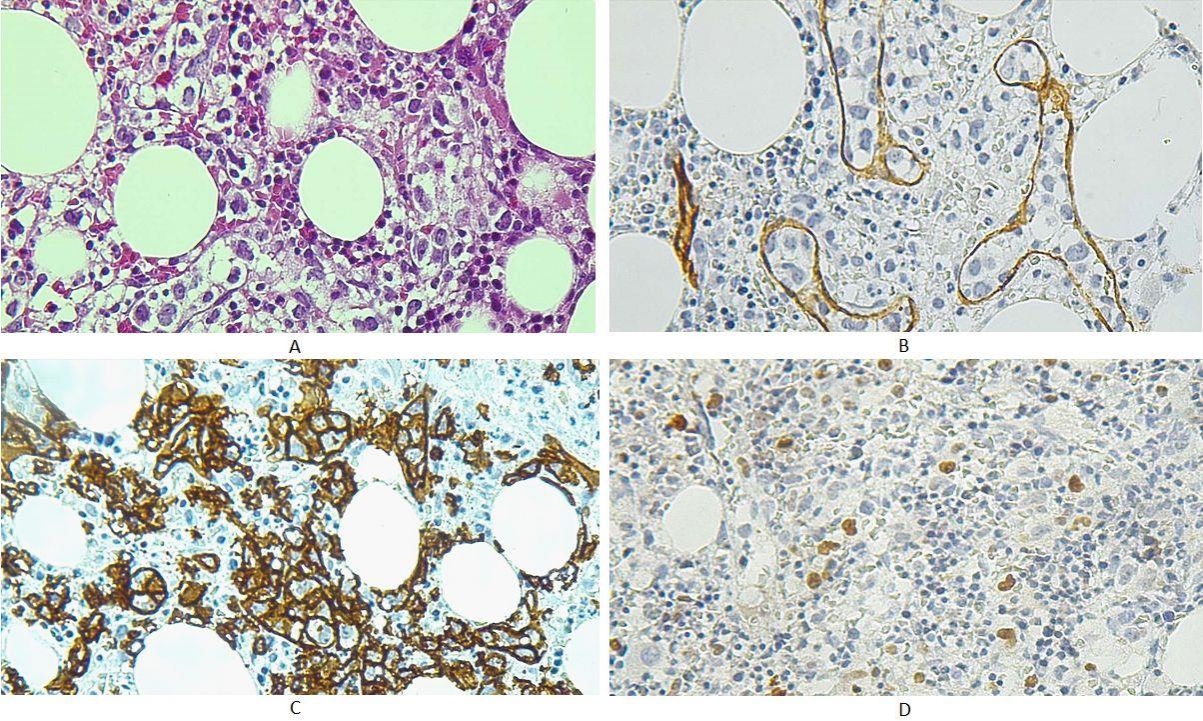
Bone marrow biopsy findings of the second patient. A. H&E stained sections show intra-sinusoidal large atypical cells infiltrate in the bone marrow spaces (x400). B. CD34 staining highlights the endothelium and intravascular growth pattern of the tumoral cells. C. CD20 staining highlights B phenotype of the lymphoid cells. D. Ki67 staining shows ahigh proliferation index of malignant cells

A 58-year-old man presented with fatigue, weight loss and bone pain for 9 months. He had a positive medical history for hypertension and diabetes mellitus. On examination, no lymphadenopathy, hepatosplenomegaly or skin rash was identified. No neurological abnormality was detected. Paraclinical investigations showed bicytopenia and increased erythrocyte sedimentation rate. The patient underwent bone marrow aspiration and biopsy twice, reported as normocellular marrow with trilineage hematopoiesis and mild increase in lymphoplasma cells. Immunohistochemical study for CD138 highlighted 10% plasma cells but staining for kappa and lambda were inconclusive. Flow cytometry was not diagnostic for multiple myeloma. Proper microbiological work-up was also performed, but no infectious etiology was found. Based on clinical findings and suspicion for multiple myeloma, he was treated with Thalidomide, but no clinical remission was observed. Therefore, he was referred to our center for further evaluation. Re-evaluation of bone marrow biopsy revealed intra-sinusoidal localization of large atypical cells with fine vesicular nuclei and prominent nucleoli. Immunophenotyping showed expression of CD20, BCL6, CD10 and BCL2, while CD34, CD3 and CD30 were associated with negative results. These findings were consistent with a diagnosis of intravascular large B cell lymphoma (Figure 3 A-D). The bone marrow aspiration was inappropriate for differential count and interpretation. No atypical cells in peripheral blood film was seen. Regrettably, his condition started deteriorating and he succumbed to the disease within 3 weeks of diagnosis.

## Discussion

These two cases, highlight the diagnostic challenges of IVLBCL, as an aggressive and rare form of large B-cell lymphoma. The pathological hallmark of the disease is the luminal proliferation of tumoral cells in small and medium-sized vessels without involvement of lymph nodes or peripheral blood (7).

The clinical presentation is varied and non-specific. The symptoms are often related to organ dysfunction due to the blood vessel occlusion, including fever, cutaneous lesions, neurological signs, hepatosplenomegaly and pancytopenia. The neurological symptoms are often significant and are due to the presence of multiple infarct sites resulting from vascular occlusion. The cutaneous manifestations are non-specific but the most common symptoms include nodular, subcutaneous, firm masses or plaques, with or without hemorrhage (2). Due to unpredictable and various clinical presentations, patients usually undergo multistep clinical investigations prior to the final diagnosis. Unfortunately, some cases are not diagnosed correctly due to the insidious intravascular growth pattern IVLBCL cells, which can be easily overlooked in routine histopathologic evaluation.

Two major forms of clinical presentation have been described for IVLBCL: 1) classical, also known as the Western form; characterized by neurological or cutaneous presentation and 2) the Asian form, which is associated with hemophagocytic syndrome (HPC), pancytopenia and multi-organ failure (1). It is suggested that concomitant presence or absence of hemophagocytosis with IVLBCL is of more value in determining the Asian form of IVLBCL, as opposed to solely designating the condition based on geographic distribution. Some studies have shown that the IVLBCL with HPC is more likely to have aggressive behavior compared to IVLBCL without HPC, regardless of geographic location (8). Both cases of this study did not present with hemophagocytosis, but with an advanced and disseminated form of the disease.

The use of cytogenetic study for IVLBCL is limited and few cases have been studied to demonstrate recurrent abnormalities. Many abnormalities have been reported in other B-cell lymphomas, including −6 or 6q− and +18, may be seen in IVLBCL but so far, no specific chromosomal alterations have been identified in IVLBCL (1,8). Unfortunately, no cytogenetic study was performed for our cases.

Most of the IVLBCL patients present with advanced, disseminated disease at the time of diagnosis. Therefore, the diagnosis of IVLBCL is often made at autopsy. Symptoms are not specific and may vary due to the infiltration of any organ (8). 

The poor prognosis is in part due to the variable presentations, which postpones the definite diagnosis. Aggressive combined chemotherapy may result in complete remission and long-term survival (2).

In both patients presented here, the clinical presentation was not specific, and the patients died shortly after diagnosis. This highlights an aggressive and subtle nature of IVLBCL, which is correlated with with the findings of previous reports. 

The origin of the IVLBCL cells has not yet been completely identified. Based on the presence of somatic mutations in immunoglobulin heavy chain variable region (VH) gene analyses, it is hypothesized that IVLBCL is originated from post-germinal center cells (1). However, other studies reported both germinal center (20%) and nongerminal center (80%) B-cell immunophenotype (9). CD10 expression has been reported in 12-13% of IVLBCL cases (1,8,10). However, there are controversial results regarding CD10 expression and different clinical outcomes (11-13). Our findings in the second patient were not supportive for a non-germinal center B cell origin (non-GCB) of tumor cells, as an immunoreactivity for both CD10 and BCL6 was found. This could be more supportive for two types of origins for neoplastic cells. CD5 expression has shown various rate of expression ranging from 22% to 75% in the previous studies (14-15). 

In a study by Murase T *et al.* on 96 cases of IVLBCL, no significant difference in clinical features or parameters were reported between non-GCB and germinal center B cell (GCB). Exclusively, they reported lower levels of CRP and higher frequency of thrombocytopenia in GCB group. Furthermore, no significant difference in prognosis was reported between CD5+ and CD5− IVLBCL patients in the study. Apparently, most of the IVBCL patients in their study were classified as non-GCB (10).

## Conclusion

In summary, we presented clinical features of IVLBCL, a rare variant of large B cell lymphoma. Cases discussed here highlight the rarity, potentially of being serious, and significance of the accurate diagnosis of IVLBCL in patients who present with progressive systemic failure with no detectable cause.

By presenting this case report, we hope clinicians and pathologists get familiar with IVLBCL and its clinical features. Early diagnosis of IVLBCL through biopsy and prompt and appropriate treatment may improve the survival rate in IVLBCL patients.
